# Molecular signature of the imprintosome complex at the mating-type locus in fission yeast

**DOI:** 10.15698/mic2018.04.623

**Published:** 2018-01-16

**Authors:** Célia Raimondi, Bernd Jagla, Caroline Proux, Hervé Waxin, Serge Gangloff, Benoit Arcangioli

**Affiliations:** 1 Genomes and Genetics department, Genome Dynamics Unit, UMR 3525 CNRS, Institut Pasteur, 25-28 rue du docteur Roux, Paris, France. Sorbonne Universités, Université Pierre et Marie Curie, Institut de Formation Doctorale, 75252 Paris Cedex 05, France.; 2 Center for Human Immunology, CRT & Hub de Bioinformatique et Biostatistiques, C3BI, Institut Pasteur, 25-28 rue du Docteur Roux, Paris, France.; 3 Genomes and Genetics department, Plate-forme Transcriptome & Epigenome, Biomics, Centre d’Innovation et Recherche Technologique (Citech), Institut Pasteur, 25-28 rue du Docteur Roux, Paris, France.; 4 Enseignement, Institut Pasteur, 25-28 rue du Docteur Roux, Paris, France.

**Keywords:** imprint, mating type switching, epigenetics, replication, Lsd1, Lsd2, Sap1

## Abstract

Genetic and molecular studies have indicated that an epigenetic imprint at* mat1*, the sexual locus of fission yeast, initiates mating type switching. The polar DNA replication of *mat1* generates an imprint on the Watson strand. The process by which the imprint is formed and maintained through the cell cycle remains unclear. To understand better the mechanism of imprint formation and stability, we characterized the recruitment of early players of mating type switching at the *mat1* region. We found that the switch activating protein 1 (Sap1) is preferentially recruited inside the *mat1M* allele on a sequence (*SS13*) that enhances the imprint. The lysine specific demethylases, Lsd1/2, that control the replication fork pause at *MPS1 *and the formation of the imprint are specifically drafted inside of *mat1*, regardless of the allele. The CENP-B homolog, Abp1, is highly enriched next to *mat1* but it is not required in the process. Additionally, we established the computational signature of the imprint. Using this signature, we show that both sides of the imprinted molecule are bound by Lsd1/2 and Sap1, suggesting a nucleoprotein protective structure defined as imprintosome.

## INTRODUCTION

**Figure 1:**
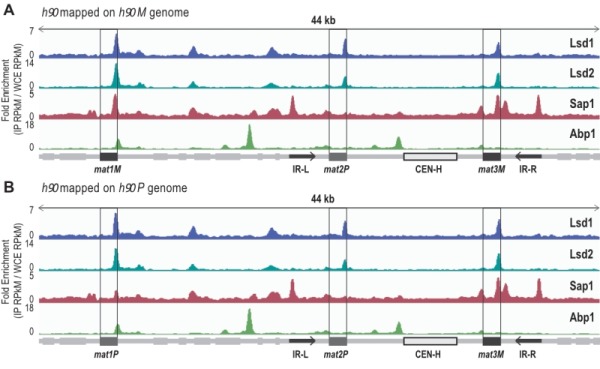
FIGURE 1: Lsd1, Lsd2, Sap1 and Abp1 are recruited to the mating type region. **(A)** Distribution of normalized enrichments of the Abp1, Lsd1, Lsd2, and Sap1 ChIPs in a *h^90^* strain (IP RPkM *reads per kilobase million*/ WCE RPkM). The sequence used for the alignment is a 44-kb region that contains the MT region with the* M* allele at *mat1*: *h^90^ M*. The 3 *mat* loci are indicated as well as the CEN-H region and the inverted repeats IR-L and IR-R. Light gray boxes show genes present in this region. **(B) **Same than in A) except that the sequence used for the alignment is a 44-kb region that contains the MT region with the P allele at *mat1*: *h^90^ P*.

Haploid *Schizosaccharomyces pombe* cells exist as two mating types (MTs), *P* (for plus) and *M* (for minus), that switch during cell divisions. The MT of the cell is determined by the allele (*M* or *P*) expressed at the *mat1* locus on the right arm of chromosome II, 500 kb away from the centromere: *mat1P* in *P* cells and *mat1M* in *M* cells 1,2. The *mat1* allele can be replaced efficiently by genetic information contained in one of the two silent donor cassettes *mat2P* and *mat3M* 2,3,4. *mat2P* and *mat3M* are located 17 kb and 29 kb centromere-distal to *mat1*, respectively 3,5, and are maintained in a silent chromatin state preventing their transcription or recombination 5,6,7. Two inverted repeats flank the donor region (*IR-L* and *IR-R*) and act as boundary elements 8,9. Additionally, a centromeric repeated sequence located between *mat2P* and *mat3M*, *CEN-H*, promotes the formation of heterochromatin (10, see Figure 1). A salient feature of the MT loci is that they are flanked by homologous sequences. The H1 homology box (59 bp) is located on the centromere distal (right) side of the cassettes and the H2 homology box (135 bp) on the centromere proximal (left) side 2. Both sequences are thought to be essential for base pairing during the initiation and resolution steps of the gene conversion process required for MT switching 11,12. The H3 box (57 bp) is located to the left of H2 at *mat2* and *mat3* 2, and is not required for MT switching 13. 

Extensive pedigree analysis at the single-cell level demonstrated that two consecutive divisions are required to produce one switched cell among four related cousins. Fission yeast possesses a remarkable genetically programmed system initiated by a site and DNA strand-specific imprint at the *mat1* locus for changing its MT (reviewed in 14,15,16). The first division produces the imprint on one of the sister chromatids at *mat1*, while the second division triggers a double-strand break (DSB) on the imprinted chromatid that initiates MT switching 17.

It was shown that the polarity of DNA replication of *mat1* is instrumental in the establishment and the strand specificity of the imprint 18. Two sites control the replication polarity at *mat1*, *RTS1* that blocks the fork coming in from the centromere and *MPS1* located near the site of the imprint that pauses the fork coming in from the right. The pausing of the replication fork at *MPS1* is a prerequisite for the formation of the imprint 14. Thus, the imprint is made on the newly synthesized lagging strand during the resumption of DNA synthesis at *MPS1* 17,19.

Molecular studies have suggested that the imprint at *mat1* is either a single-strand DNA break 20 or one or two ribonucleotides 21. The imprint was mapped to the Watson strand corresponding to the neo-synthesized lagging strand, at the junction of the *mat1* allele and the H1 homology box 22. Furthermore, the position of the imprint is site-specific but sequence-independent 19,20. Interestingly, the position of the break on the Watson strand at *mat1* differs by three nucleotides between the *mat1P* and *mat1M* alleles 22, indicating that the position is dictated from within the *mat1M* and *mat1P* sequences. The imprint is protected against DNA repair and remains stable throughout the entire length of the cell cycle to be transiently converted to a polar DSB during the following S-phase 17,20. Repair of this break is not random; *mat1P* prefers *mat3M*, and *mat1M* prefers the nearby *mat2P* in 90% of switches. This preference is called directionality of switching 23. In strains in which the donor sequences are deleted 24, the position and level of the imprint are identical to that in wild type, but the DSB is repaired off the sister 24,25.

Several *cis-*acting sequences are required for the formation and maintenance of the imprint. The SS2 and SS13 deletions are located within *mat1M*. SS2 is important for the pause and the imprint, whereas SS13 acts as a spacer element that allows efficient imprinting 19. To the right of *mat1M*, the *Msmt-0* deletion maintains efficient pausing but abolishes the imprint 18,26. The Lsd1/2 (Lysine Specific Demethylases) and the Swi1/Swi3 (Switch gene) complexes are necessary for the pause and the subsequent imprint 17,27,28. Lsd1 is part of a complex including Lsd2 as well as Phf1/Phf2, two plant homeodomain finger proteins 29. The complex also associates with topoisomerase 2 (Top2), replication factor A (Rfa1) and Sap1 29, essential proteins with a role in DNA replication. The involvement of Lsd1 in DNA replication and DNA damage response is not new 30, and we showed that Lsd1/2 act redundantly to promote the pause through both their HMG (high mobility group) domain and amine oxidase activity. In addition, the recruitment of Lsd1/2 to *mat1* requires SS2 27. Swi1/3 proteins are associated with the replication fork and stabilize it during the pause 28,31. Sap1, the Switch Activating Protein 1 is an essential gene that interacts with SAS1, a sequence covered by the *Msmt-0* deletion 32. Sap1 was also described to play a role in DNA replication 33,34,35,36, retrotransposition targeting 37,38 and chromosomal organization 39,40. Finally, Abp1 (ARS Binding Protein 1), a CENP-B (Centromeric Protein B) homolog that antagonizes Sap1 at LTRs (long terminal repeat), is enriched next to *SAS1*, to the right of *mat1M* 35. Abp1 was proposed to control the directionality of switching by regulating the alternative expression of the *swi2* gene (Switch gene 2) 41,42,43, important for the spreading of the Swi2/Swi5 recombination-promoting complex on the* mat2/3* region 43.

To advance our understanding of the process of imprint formation and stability, we analyzed by chromatin-immunoprecipitation and whole genome sequencing (ChIP-seq) the recruitment of Abp1, Lsd1, Lsd2, and Sap1 proteins to the *mat1* region. Because the Lsd1/2 complex interacts with a *cis*-acting element located within the MT cassettes and promotes replication pause at *mat1* but not at *mat2P* and *mat3M*, we compared the interactions of the four proteins in the wild-type strain and in two strains containing a deletion of both the *mat2P* and *mat3M* cassettes. Thus, we have established the computational signature of the imprint and propose a model in which Lsd1 and Lsd2 are exclusively recruited to the *mat1 *locus.

## RESULTS

### ChIP-sequencing mapping of Abp1, Lsd1/2 and Sap1 to the *h^90^*mating type region

It has been previously reported that Abp1, Lsd1/2 and Sap1 bind to the *mat1* region 27,35,44. However, several limitations arose from those studies: The strains that were initially used to map Abp1 and Sap1 by ChIP-seq were rearranged in the MT region (h^-S^), and the length of the reads (30 nucleotides) prevented their unambiguous assignment to *mat1, 2 or 3 *cassettes 35. Concerning Lsd1/2, whole genome analyses had been achieved by ChIP-chip 45, but no detailed analysis of the MT region was performed. More recently, we mapped Lsd1/2 to *mat1* by ChIP-qPCR 27.

To extend the mapping of these proteins to the entire MT region, we performed ChIP-seq of Abp1, Lsd1/2 and Sap1 on the wild-type and switching proficient *h^90^* strain (Figure 1). To achieve proper mapping, we constructed two reference genomes carrying either the *M* or *P* allele at *mat1* (Figure 1). We found that both Lsd1 and Lsd2 are enriched at the *mat1*, *2* and *3* cassettes, whereas Sap1 was more enriched at the *mat1M *and *mat3M* cassettes than *mat1P* and *mat2P *(Figure 1). Additionally, Sap1 was found to be enriched at the IR-L and IR-R boundary elements 8,46. Concerning Abp1, we confirmed that it is indeed enriched at the left border of *CEN-H* 41,47. Interestingly, we found that Abp1 is also enriched at the right border of *mat1* in an *h^90^* background. This observation prompted us to investigate the potential involvement of Abp1 in the early steps of MT switching.

### Abp1 is recruited to *swi2* when *M* is present at *mat1*

**Figure 2:**
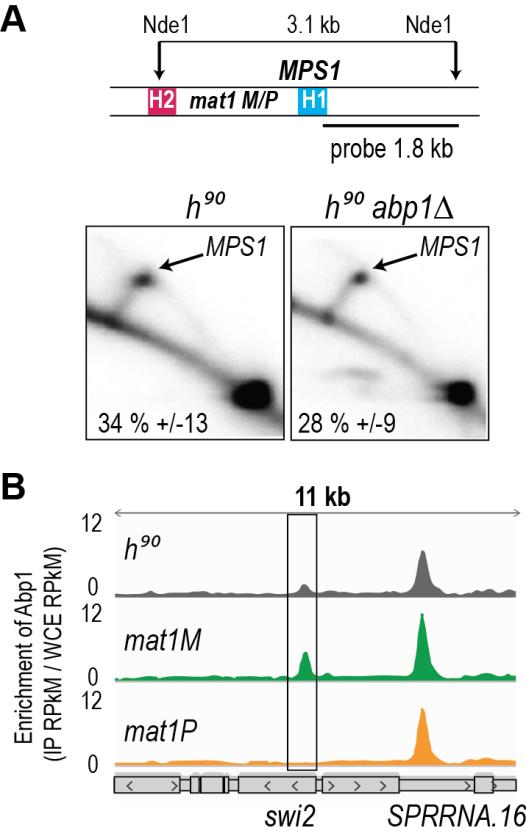
FIGURE 2: Abp1 does not control *MPS1* but is recruited at *swi2 *as a function of the mating type. **(A)** The upper panel indicates the probe and restriction enzyme used for the analysis of the replication intermediates. The lower panel represents the 2D gel analysis of the *MPS1* site at *mat1 *in *h^90^* strain and in *h^90^ abp1∆* strain (n=3). **(B)** Distribution of normalized enrichments of the Abp1 ChIPs at the *swi2* locus (IP RPkM / WCE RPkM) in *h^90^*(gray), *mat1M* stable (green) and *mat1P* stable (orange). Gray boxes indicate the genes in the region and arrows the transcription direction. Loci where Abp1 is enriched are annotated.

Using 2D gel analysis, we addressed the role of Abp1 in *MPS1* activity. Unlike the situation observed at LTRs, Abp1 is not required for the pausing activity of *MPS1* (Figure 2A), consistent with Abp1 being dispensable for the formation and maintenance of the imprint 41. Earlier work has shown that Mc, encoded from the *mat1M* cassette, and Abp1 control the expression of Swi2, a key player in the directionality process 42,43. Moreover, the recruitment of Mc to *swi2* depends on Abp1 42. Reciprocally, using the two stable* M *and* P* strains with deleted donor sequences, we found that the enrichment of Abp1 at *swi2* is restricted to* M* cells, while the enrichment at the neighboring peak is unaffected by the MT of the cells (Figure 2B). The genome wide analysis showed that Abp1 M-specific interaction is unique for the *swi2* gene.

### Lsd1/2, Sap1 and Abp1 map at *mat1*

**Figure 3:**
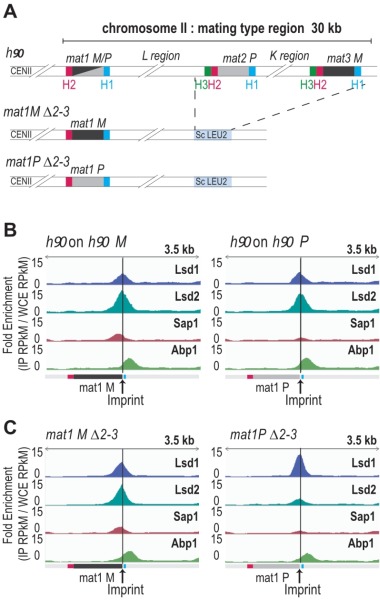
FIGURE 3: Abp1, Lsd1/2 and Sap1 are recruited at *mat1.* **(A)** Schematic view of the strains used. The stable *mat1M *∆*2-3* and *mat1P *∆*2-3* strains as well as the wild type *h^90^* strain are drawn. **(B)** On the left: distribution of normalized enrichments of the Abp1, Lsd1, Lsd2, and Sap1 ChIPs on the *h^90^* background (IP RPkM / WCE RPkM). The sequence used for the alignment is a 44-kb region that contains the MT region with the* M* allele at *mat1*: *h^90^ M*. Only *mat1M *is shown. On the right: same as at the left, with the *h^90^* P sequence used for the alignment. Only *mat1P *is shown. **(C)** Same as in (B) with the *mat1M *∆*2-3* background on the left and the *mat1P *∆*2-3* background on the right. The sequence used for the alignments is a 10-kb region containing *mat1M* (for the left panel) or *mat1P *(for the right panel). (B-C) the imprint site is indicated as well as the H2 (pink) and H1 (blue) homology boxes.

The enrichment positions of the four proteins and the repeated nature of the MT cassettes introduce an ambiguity that cannot be resolved during alignment. To pinpoint the Lsd1/2 and Sap1 binding sites to *mat1*, we used the *mat1M *∆*2-3* and *mat1P *∆*2-3* strains deleted for the donor sequences 24 (Figure 3A). Abp1 is enriched outside of the *mat1* locus (to the right of the H1 cassette), and its enrichment is independent of the allele present at *mat1* (Figure 3B, 3C). Lsd1 and Lsd2 are enriched both in *mat1M* and *mat1P* with Lsd2 and Sap1 more enriched in *mat1M* (Figure 3B, 3C). 

**Figure 4:**
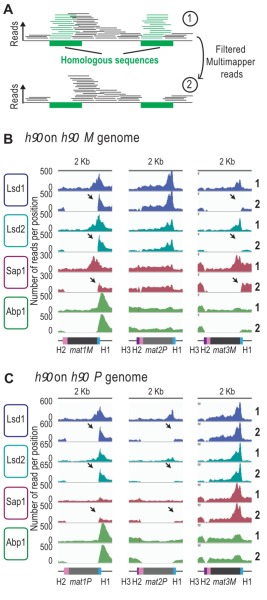
FIGURE 4: Lsd1/2 and Abp1 are recruited only at *mat1*. **(A)** Schematic representation of the analysis used. Green boxes represent homologous sequences. Reads that can be unambiguously mapped are represented by black lines and multi-mapper reads are represented by green lines. Upper drawing represents the result of an alignment without filter (1). Lower drawing represents the alignment after that multi-mapper reads are removed using "samtools" algorithm (2). **(B)** Distribution of raw coverage of the Abp1, Lsd1, Lsd2, and Sap1 ChIPs. The sequence used for the alignment is a 44-kb region that contains the MT region with the* M* allele at *mat1*: *h^90^ M*. The lines numbered 1 are the coverage without filter and the lines numbered 2 are the coverage obtained after removal of multi-mapper reads. The arrows indicate the unique-mapper reads at the junction of the *mat* loci. **(C)** Same as in (B) with the *h^90 ^P *sequence used for the alignment.

We next analyzed the reads that can only map to a unique position on the reference genome (unique mapper) in a *h^90^* wild type context (Figure 4A), where the donor sequences are present (Supplementary Figure 4). As expected, the highly repetitive sequences of IR-L and IR-R are no longer covered in the two *h^90^* genomes (*h^90^* *M* and *h^90^* *P*) (Supplementary Figure 4A and 4B). When the *M* allele is at *mat1*, we found no unique mapper reads at *mat1* and *mat3* (Figure 4B). Similarly, when the *P* allele is at *mat1*, no unique mapper reads at *mat1* and *mat2* were found (Figure 4C). Conversely, Abp1 is located outside of the *mat1* cassette in a unique region and can thus be used for alignment. In the case of Lsd1/2, the enriched unique mappers are well maintained at *mat1* (both in the *M* and *P* context) and strongly diminished to nearly background level at*mat2P* and *mat3M* loci, indicating that Lsd1/2 are preferentially recruited at *mat1*. Furthermore, the absence of Lsd1/2 at the two silent loci was confirmed using ChIP-qPCR (Supplementary Figure 4C). The situation for Sap1 is less clear, and its profile suggests that it is recruited to *M* but poorly to *P*. This hints that Sap1 is recruited both inside *mat1M* and to SAS1 outside of *mat1*.

### Characterization of the Sap1 binding in the *M* allele

**Figure 5:**
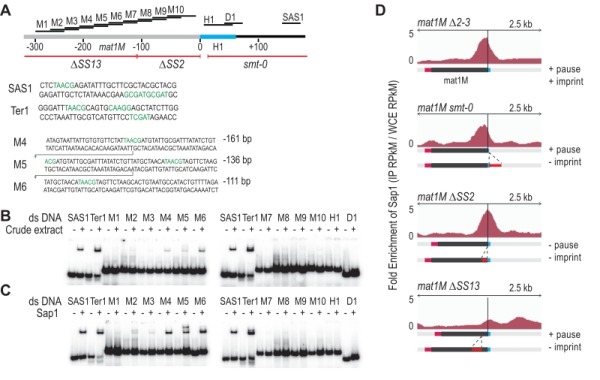
FIGURE 5: Sap1 is recruited in *mat1M*. **(A)** Schematic view of right region of the *mat1M* locus. The probes used for the shift assay as well as the sites of the deletion strains used for the ChIP-seq of Sap1 are indicated. The sequence of the probes that trigger a shift in B and C are indicated. **(B)** Electrophoretic mobility shift analysis (EMSA) of crude extracts from *S. pombe*. *SAS1* and *Ter1 *are used as controls. In lanes (-), 1 ng of radio labelled dsDNA is used with no proteins. In lanes (+), 5 µg of total proteins and 1 ng of radio labelled ds DNA are used. **(C)** EMSA of Sap1-6xHis protein purified from *E. coli*. In lanes (-), 1 ng of radio labelled ds DNA is used with no proteins. In lanes (+), 40 ng of Sap1-6xHis protein and 1 ng of radio labelled ds DNA are used. **(D)** Distribution of normalized enrichments of Sap1 ChIPs (IP RPkM / WCE RPkM) in the* mat1M *∆*2-3, mat1M smt-0 *∆*2-3, mat1M *∆*SS2 *∆*2-3 *and *mat1M *∆*SS13 *∆*2-3 *backgrounds. The sequence used for the alignment is a 10-kb region that contains the *mat1M*, *mat1M smt-0, mat1M *∆*SS2* or *mat1M *∆*SS13*, respectively*. *Only *mat1M *is shown. The MT switching phenotype of the deletion strains is indicated.

We used *in vitro* and *in vivo* complementary approaches to further determine Sap1 binding sequences in the *M* allele. The gel shift experiments were achieved using 14 double-stranded DNA probes labeled in 5'. Two positive controls were included (*SAS1* and *Ter1*) in addition to 10 regions mapping inside the *M* allele and 2 mapping outside (Figure 5A). Using crude protein extracts, we observed the expected mobility shift for *SAS1* and *Ter1* as well as for 3 probes inside the M allele (Figure 5B). We used purified Sap1 protein to confirm that it is responsible for the shift (Figure 5C). Since M4, M5 and M6 are part of a region involved in the stability of the imprint (SS13) 19, we analyzed by ChIP-seq the enrichment of Sap1 in several donor less strains with deletions spanning the *mat1M* locus. The *Msmt-0* deletion does not change the enrichment signal within *mat1M*, whereas the SS13 deletion strongly decreases the Sap1 signal, supporting the *in vitro *interaction result (Figure 5D *Msmt-0* and SS13). The slight decrease on the right of the *mat1M smt-0*-containing deletion together with the remaining weak Sap1 signal in SS13 is attributed to the SAS1 interaction located 140 bp away from the imprint. The SS2 deletion abolishes DNA replication pausing and imprinting 19 but does not affect Sap1 enrichment, whose signal is slightly shifted toward the imprint (Figure 5D ∆SS2). These results indicate that Sap1 is preferentially enriched *in vivo *on two sequences (SS13 and SAS1) that are not necessary for replication fork pausing but required for imprint formation or maintenance, at least at the *mat1M* locus.

### Detection of an imprinted site by high throughput sequencing

**Figure 6:**
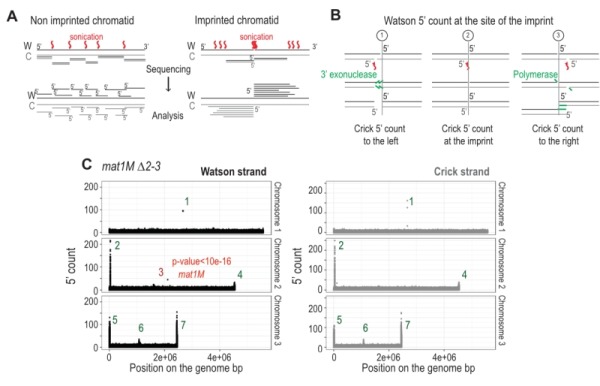
FIGURE 6: The imprint site is detectable by high throughput sequencing on the Watson strand. **(A)** Schematic view of the result of the sonication of both chromatid types. Red arrows indicate sites of sonication breakage. The black strand is the Watson strand (W) and the gray the Crick (C). 5' of Watson are shown in black and those of Crick in gray. Lower panel indicates the different types of reads obtained after sequencing. **(B)** Schematic representation of the blunting of the end that is used during libraries preparation. The Crick and Watson 5' ends are preserved after treatment. The Crick strand can break at 3 positions compared to the imprint site: to the left, across or to the right. **(C)** Distribution of the 5' count per position in the genome on both strands. *mat1M *∆*2-3* stain was used for the analysis. p-value is calculated using a theoretical t-student distribution that fits on the distribution of the whole-genome observed 5' count. Regions that have a high 5' count are numerated.

**Figure 7:**
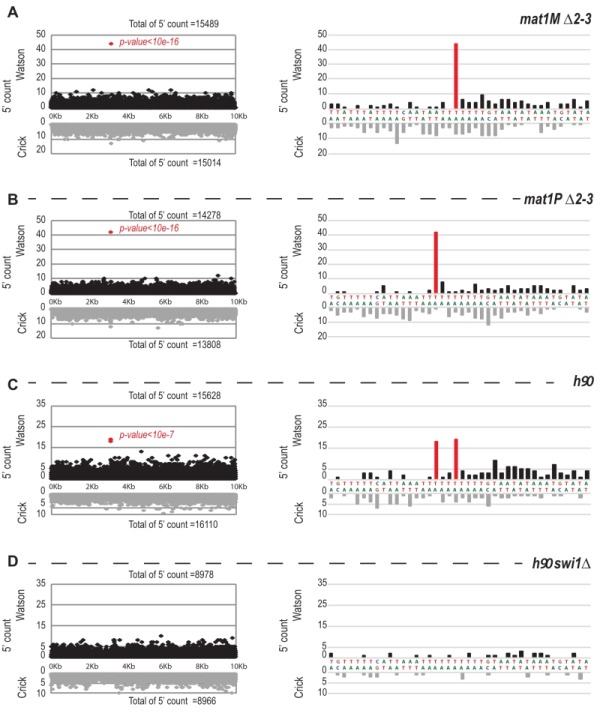
FIGURE 7: 5' count on the Watson strand is biased precisely at the site of the imprint. On the left: distribution of the 5' count of the Watson strand (black) and the Crick strand (gray) on the 10-kb region containing *mat1*. On the right: zoom on the imprint site. Red bars represent the 5' count enriched at the imprint sites. p-value is calculated using a theoretical t-student distribution that fits on the distribution of the whole-genome observed 5' count. **(A)** The sequencing of *mat1M *∆*2-3* strain is represented, **(B)** *mat1P *∆*2-3 *strain, **(C)** *h^90^* strain and **(D)** *h^90^* *swi1*∆ strain. ABCD) The *mat1M *∆*2-3*, *mat1P *∆*2-3* and *h^90^ P* genomes are used, respectively.

The imprint was described as either a single stranded break 20 or a one/two ribonucleotide(s) 21,48 that behave(s) as a mechanical fragile site 49. Because the chromatin preparation and the Illumina sequencing steps will reveal the imprint as a single strand break, we portray the imprint as a single strand break in the remainder of the text. In Figure 6A, we reasoned that upon chromatin sonication, the strand opposite to the nick will preferentially break at its vicinity. Library preparation using the Illumina kit (TruSeq Chip) uses an exonuclease and a DNA polymerase to process the ends to achieve efficient ligation prior to sequencing (Figure 6B). This method conserves the first nucleotide of the 5’ end of all sonicated fragments. Consequently, reads starting at the imprint are expected to be more frequent in the whole chromatin extract even prior to any chromatin immunoprecipitation. Furthermore, it is possible to count for each DNA strand (Watson and Crick) the number of 5' ends at each position in the genome, and we will refer to this count as "5' count" in the rest of the manuscript (see 5’ count analysis in Materials and Methods). A whole genome distribution of the 5’ count was established on the *mat1M* ∆*2-3* (Figure 6C) and *mat1P* ∆*2-3* and *h^90^* strains (Figure 7B-C) using 130 nucleotide-long reads. 7 enriched regions were detected on the Watson strand (Figure 6C). 6 such regions were also detected on the Crick strand (Figure 6C). They delineate either repeated regions or regions exhibiting homology with the mitochondrial genome (Supplementary Table 1). The last enriched 5’ count is only found on the Watson strand and corresponds to the imprint site at *mat1* (Figure 6C, in red). 

We next focused on the 10-kb region surrounding the *mat1* locus (Figure 7). The overall distribution of 5’ count is similar on the Watson and Crick strands, except for one nucleotide (in red) significantly over-represented on the Watson strand (Figure 7). The position in *mat1M* ∆*2-3* and *mat1P* ∆*2-3* corresponds to the imprinted position that is 3 nucleotides apart in *M* and *P* 22 (Figure 7A and 7B). As expected, both positions are enriched in 5' count in the *h^90^* background that contains a mixture of *mat1M* and *mat1P* alleles (Figure 7C). To confirm that the signal results from the imprint, we used the *swi1*∆ mutant strain that exhibits a low level of imprint. In this genetic background, no nucleotide shows any bias in 5' count (Figure 7D).

**Figure 8:**
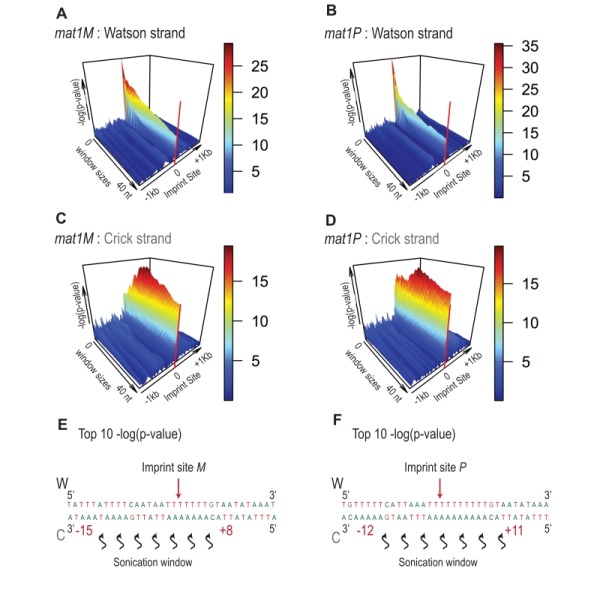
FIGURE 8: Signature of sonication across the imprint. Statistical analysis of the 5' count in the *mat1* region. -log(p-value) of 5' count as a function of the window size and genome position is represented. The -log(p-value) scale is indicated on the right of the graphics. The p-value is calculated using theoretical t-student distributions that fit with the distribution of 5' count observe in the 10-kb region that contains *mat1*. **(A)** A sliding window (+/- 1 kb around the imprint site) with step size one and varying window size (1 to 40 nt) is applied when calculating the -log(p-value) of the 5' count of the Watson strand shown. The sequencing of a *mat1M *∆*2-3* strain is used. **(B)** Same as in (A) for the Crick strand. **(C)** Window sizes and window positions compared to imprint site for the top 10 -log(p-value) are indicated. **(D, E and F)** Same as in (A, B and C), respectively for the sequencing of a *mat1P *∆*2-3* strain.

Because the bias in 5’ count is only visible on the imprinted strand, we conclude that sonication does not preferentially break the phosphate bond facing the nick. Therefore, we searched on the Crick strand for a region with a strong 5’ count statistical bias. Using a sliding window with different window sizes, we calculated the p-value in the 10-kb region surrounding *mat1* (Figure 8). As expected, the window of one nucleotide at the site of the imprint has the lowest p-value on the Watson strand (Figure 8A and 8B). On the Crick strand, the most significant p-value is contained within a window of 17 to 23 nucleotides facing the imprint (Figure 8C and 8D). This corresponds to the molecular signature of a confined and persistent single-strand break upon chromatin sonication: A unique nucleotide on one strand and a larger region on the opposite strand (Figure 8E and 8F).

### Immunoprecipitation of the imprinted chromatid

**Figure 9:**
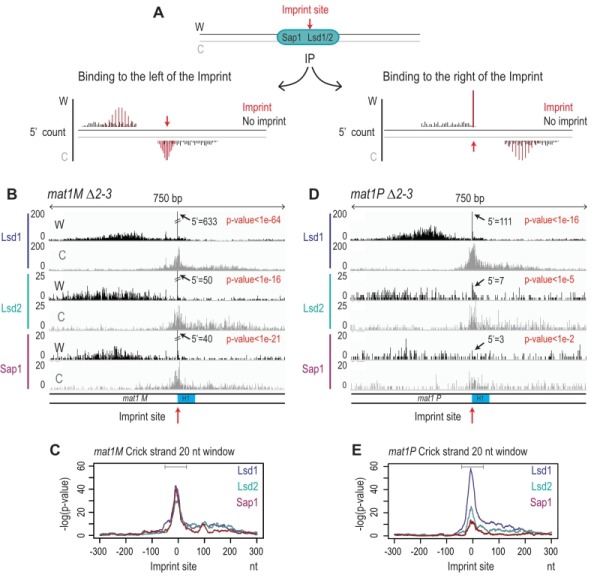
FIGURE 9: Lsd1/2 and Sap1 immunoprecipitate chromatids on both sides of the imprint. Result of the 5' count analysis of the ChIP data presented in Figure 3C. **(A)** Schematic representation of the theoretical 5' count distribution of Lsd1/2 and Sap1. In black, the 5' count of a non-imprinted chromatid and in red that of an imprinted chromatid. **(B)** Distribution of the 5' count of the Lsd1/2 and Sap1 ChIPs for the Watson strand (black) and Crick strand (gray) in the *mat1M *∆*2-3* background. The 5' count at the imprint site is indicated. The p-value is calculated using theoretical t-student distributions that fit with the distribution of 5' count observed in the 10-kb region that contains *mat1*. **(C)** Analysis of the 5' count bias on the crick strand using a 20 nt window in a *mat1M *∆*2-3* strains for the IPs of Lsd1-2 and Sap1. A sliding window of 20 nt with set size one is used in a 10 kb region around *mat1 *is applied. Gray line indicates the bias of the crick across the imprint. **(D)** Same as in (B) in a *mat1P *∆*2-3* strain. **(E)** Same as in (C) in a *mat1P *∆*2-3* strain.

To determine whether Lsd1, Lsd2, and Sap1 interact with the imprinted chromatid and on the right or left of the imprint we used the molecular signature defined above. Figure 9A shows the expected distributions of enriched 5’ count upon immunoprecipitation. Since Lsd1/2 and Sap1 bind the repeated *mat* cassettes, we circumvented the alignment problem by performing the experiment in the donor less strains. For Lsd1, the enrichment of the 5' count at the imprint on the Watson and Crick strands (Figure 9B) at *mat1M* exhibits the molecular signature defined above (Figure 9C). This result shows that Lsd1 interacts on both sides or directly with the imprinted chromatid. The additional spreading of the 5’ count on both DNA strands indicates that Lsd1 also interacts with the chromatid in the absence of imprint (Figure 9B). A similar 5’ count distribution is observed for Lsd1 at *mat1P *(Figure 9D and 9E). Lsd2 and Sap1 exhibit a similar pattern at *mat1M *as well, while their interaction is weaker at *mat1P* (Figure 9D and 9E). Altogether, our results indicate that Lsd1, Lsd2 and Sap1 interact on both sides of the imprint but also with *mat1* regardless of the imprint. Intriguingly, the interaction at* mat1P* is weaker for Lsd2 and Sap1.

## DISCUSSION

In this work, we propose a molecular definition of the imprint upon sonication and genomic sequencing. Our computational analysis demonstrates that a site- and strand-specific persistent imprint/nick can be unambiguously mapped. The computation of the 5’ count reveals a unique accumulation at the site of the imprint on the Watson strand, whereas the opposite Crick strand is mechanically broken by sonication within a window of 20 nucleotides, thus yielding a more diffuse accumulation of 5’ ends. This molecular signature is consistent with the free rotation of the Crick strand that generates a population of molecules with weakened A-T rich Watson-Crick pairing on both sides of the nick and makes the resulting single-strand in this window highly prone to breakage. However, we cannot rule out that the proteins preventing the imprint from being repaired contribute as well to the pattern of shearing of the Crick strand.

We also provide data for the chromosomal enrichment at the genome wide level of four proteins, Lsd1, Lsd2, Sap1, and Abp1. Abp1 is strongly interacting with the sequences near *mat1*, but a function in replication fork pausing and imprinting was ruled out. The M-specific enrichment of Abp1 next to the Mc binding site located in the *swi2* gene reinforces the role of Swi2 in directionality 42,43. 

In mammals, Lsd1 plays an important role in gene expression by establishing and maintaining proper epigenetic marks. Several layers control the chromosomal targeting of Lsd1 for positive or negative transcription regulation. CoREST targets the Lsd1 complex to H3K4 50, whereas the human androgen receptor targets Lsd1 to H3K9 51. The plant homeodomain finger protein BHC80 and the HMG protein Braf35 are part of the core complex Lsd1-CoREST (reviewed in 52). BHC80 participates to the reading of histone marks 53 and Braf35 54 is a structural DNA-binding protein.

In fission yeast, several pieces of evidence support the specific demethylation of H3K9 by Lsd1 at many promoters and boundary elements, but its role on H3K4 was not established 29,55. Lsd1 is part of a complex including Lsd2 and Phf1/Phf2, two plant homeodomain finger proteins 53, and Lsd1 and Lsd2 are both fused to an HMG domain in fungi 56. Taken together, the protein composition of the Lsd1/2 core complex is quite similar in fission yeast and mammals and the main functional difference resides in the absence of H3K4 demethylation. Because Sap1 interacts with the Lsd1/2 complex 29 and that Sap1 and Lsd1/2 complex are overlapping at several locations within the genome (see Supplemental Figure 1A) and notably at highly expressed genes (see Supplemental Figure 1B-E), we propose that Sap1 participates in the targeting of the Lsd1/2 complex to prevent H3K9 methylation at key promoters and therefore define heterochromatin boundaries 29,57. 

The situation is more complicated at the mating type locus, where the methylation state of H3K9 is not involved in the early mating type switching steps. At this locus, the presence or absence of the sole H3K9 methyltransferase Clr4 does not rescue the mating type switching defect observed when *lsd1* is mutated 27. Although *mat1P* and *mat1M* exhibit no sequence homology, a remarkable finding is that Lsd1 and Lsd2 are enriched in both alleles on sequences that are necessary to promote replication fork pauses at *MPS1* on the chromosome, and sufficient on a plasmid system 19. Interestingly, when *P* and *M* sequences are present at the silent *mat2P* and *mat3M* loci, Lsd1 and Lsd2 are not detected (Supplemental Figure S2), consistent with the absence of replication fork pause at these loci 19. In addition, the absence of H3K9 methylation (*clr4* mutant) is not sufficient to trigger recruitment of Lsd1/2 at the silent *mat2/3* loci (data not shown). We propose that the Phf1/Phf2 histone code readers participate to the targeting of Lsd1/2 at *mat1* and probably at the other transcriptionally active loci 53.

By using the molecular signature of the imprint, we provide strong evidence that the Lsd1/2 complex is interacting at the imprint site, prior and after imprint formation. The same conclusion was reached by showing that Lsd1/2 is working upstream of Swi1/3 and is still enriched at *mat1 *in the absence of an imprint 27. The two Sap1 binding sites on either side of the imprint (SS13 and SAS1) overlap with regions that are dispensable for both replication fork pausing and Lsd1/2 initial recruitment, but are essential for imprint formation or maintenance, at least when *mat1* contains the M allele. When *mat1* contains the P allele, only the SAS1 site remains, since no enrichment for Sap1 could be detected in the P sequences. This result further indicates that the processes required for replication fork pause and imprinting formation/protection are not identical in M and P cells. One possibility would be that Sap1 is either modified and not efficiently recognized during the immunoprecipitation or replaced by another protein in P cells.

**Figure 10:**
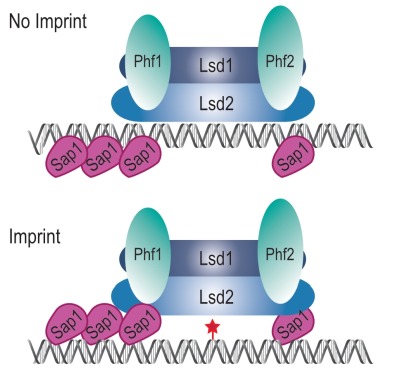
FIGURE 10: The imprintosome. Schematic view of the model of binding of Lsd1/2 and Sap1 at *mat1*. Lsd1/2 complex is represented in blue with Phf1/2 in green. Sap1 is represented in pink. The imprint is represented by a red star. Upper panel, Lsd1/2 complex is recruited on the imprint site on the chromatid with no imprint independently of Sap1. Lower panel, when the imprint is formed Sap1 stabilizes the Lsd1/2 complex.

The simplest model accounting for our results is that the Lsd1/2 complex is epigenetically recruited to *mat1* independently of Sap1 to promote replication fork pausing at *MPS1 *(Figure 10, No Imprint). Following replication fork restart and imprinting, Sap1 and Lsd1/2 form a chromatin structure, the imprintosome, that protects the imprint from being repaired (Figure 10, Imprint). We propose that Lsd1/2 controls replication fork pausing at *mat1* on both the imprinted and the non-imprinted chromatids, thus allowing the fork to restart in the same environment at each round of DNA replication and produce persistently and efficiently the imprint.

## MATERIALS AND METHODS

### Fission yeast strains and genetic procedures 

The *S. pombe* strains used in this study are listed in Supplemental Table S3. Standard media (YES) and genetic protocols for fission yeast were used 58.

### 2D Gel Analysis

2D gel analysis of replication intermediates was carried out as described previously 59. DNA was prepared and digested in agarose plugs with the indicated restriction enzymes 12. Enriched fractions for replication intermediates were obtained using BND cellulose columns (SIGMA). Gels were blotted onto Hybond-N+ nylon membrane. The probes were labeled with alpha-^32^P dCTP and the blot analyzed on a PhosphoImager and quantified with the ImageQuant software (pause/ arc + pause in percentage) (GE Healthcare Life Sciences). Sequences of primers used to produce the probe are indicated in Table 2.

### Gel shift assay

Production of Sap1-6His by *E. coli* was induced with 1 mM IPTG as previously described 32. Sap1-6His was purified using column of Ni-NTA His-tag from Qiagen according to the manufacturer’s instructions. Sequences used are listed in Table 2. 1.50 ng of a given oligonucleotide was labeled with gamma-^32^P with T4 polynucleotide Kinase (NEB) and incubated with its cold complementary sequence. 1 ng of the resulting double stranded probe was incubated with 5 µg of total protein extract or with 40 ng of purified Sap1-6His 44. Gel shift was performed as previously described 60.

### Chromatin Immunoprecipitation (ChIP)

ChIP-sequencing was performed as previously described 35 with the following modifications: fixation for ChIP of Lsd1 and Lsd2 was carried out at 25°C for 20 min with 1% of paraformaldehyde (PFA) and fixation for ChIP of Sap1 and Abp1 was performed at 18°C for 15 min with 3% of PFA. For Lsd1 and Lsd2, an Anti-Myc-Tag mAb-magnetic Beads (M047-11 MBL) was used. For Abp1 and Sap1, Dynabeads Protein G 10007D (Invitrogen) coupled with Tap Tag antibody CAB1001 (Pierce Thermo) or with polyclonal serum against the native Sap1^53^, respectively, were used. For the ChIP followed by qPCR analysis, 3x10^8^ cells were used with one tenth the amount of reagents.

### quantitative PCR (q-PCR)

qPCR was performed using a master mix (Eurobio Green qPCR Mix Lo-Rox) on the qTower Analytik Jena AG A6 machine. Each value is the mean of technical triplicates. The enrichment was obtained using the ∆∆Cq normalization method using the value of a pair of primers localized 3 kb away from *mat1 *and the WCE values. The list of primer sequences is in Supplemental Table S4. Experiments were performed 3-times and unpaired t-test were used to calculate the p values.

### Illumina Sequencing of DNA

Libraries were prepared using TruSeq ChIP Library Preparation kit following the manufacturer’s instructions (Illumina). Libraries are checked for concentration and quality on DNA chips with a Bioanalyser (Agilent). The libraries were quantified by fluorimetric measurements with the Qubit® dsDNA HS Assay Kit (ThermoFisher). 65-bp and 130-bp Single Read sequences were generated on the Hiseq2500 sequencer according to manufacturer’s instructions (Illumina). Library statistics are provided in Supplemental Table S5.

### Illumina Read Processing and Alignment of DNA Libraries

Illumina reads were aligned to the *S. pombe* genome assembly ASM294v2.23 using Bowtie v2.1.0 61 with default parameters and multi-mapper reads randomly distributed. Using BamTools 62, read counts were normalized to reads per million per kilo base (RPKM), using the total library size and a bin size of 1 kb. For the analysis of multi-mapper reads, we filtered aligned read files with samtools 63 using the parameter -q 10.

### ChIP-Sequencing Analysis

ChIP peaks versus appropriate inputs (whole-cell extracts) were called using MACS v2.1.0 64. Statistic validation of peaks was performed using the IDR method with ENCODE recommendations 65. Genome browser tracks and meta-analysis were created using enrichment (IP reads per million (RPM) / input RPM) of indicated sequencing. Intersection of lists of peaks were calculated with BEDTools v.2.17.02 66 (parameters: -u). For the transcriptional analysis 2 groups of 108 genes were defined and intersected with the list of peaks shared by Lsd1/2 and Sap1. The first group contains the 108 most present transcripts and the 108 less present transcripts using the normalized data from 67.

### 5' count analysis

We used a script to extract the beginning of reads that map on the Watson or Crick strands. Enrichments of 5' count were obtained using the total library size. p-values were obtained using the negative binomial distribution fitting function in R.

All scripts used for the analysis and outputs are available upon request.

## SUPPLEMENTAL MATERIAL

Click here for supplemental data file.

All supplemental data for this article are also available online at http://microbialcell.com/researcharticles/molecular-signature-of-the-imprintosome-complex-at-the-mating-type-locus-in-fission-yeast/.
