# Supplementary material for: Molecular signature of the imprintosome complex at the mating-type locus in fission yeast
Source: Microb Cell. 2018 Jan 16;5(4):169–83. doi: 10.15698/mic2018.04.623 (PMC5878685; doi:10.15698/mic2018.04.623)
Supplement: Supplementary file 1 [file mic-05-169-s01.pdf]

SUPPLEMENTAL MATERIAL AND METHODS

Processing of sequencing data

Reads obtained using the illumina technology (see MM) were aligned using the bowtie2 algorithm [61] with the genome of *S. pombe* (ASM294v2.23) and with several contigs depending on the genotype of the strain. The strains h^{90} were aligned with contigs that contain 45 kb of the wild type MT region with the *M* allele at *mat1* (file named h90M-45.fa) or with the *P* allele at *mat1* (file named h90P-45.fa). Strains *mat1M Δ2-3* were aligned with a contig that contains the 10-kb region with *mat1M* (file named mat1M.fa) and strains *mat1P Δ2-3* were aligned with a contig that contains the 10 kb region with *mat1P* (file named mat1P.fa). Strains containing deletions at *mat1M* were first aligned with mat1M.fa contigs and next with contigs deleted for the appropriated sequence (file named mat1MSS2.fa, mat1MSS13.fa and mat1Msmt0.fa).

Next, the normalized coverage was calculated using bamtools package version 2.2.3 [62] (parameters: --normalizeUsingRPKM --binSize 1). The bigWig file was converted to Wig files with bedtools v.2.17.0 and BigWig_tools.v4 [66, 68]. IGV genome browser was used to normalize IP coverage with WCE coverage, using combined data track we divide the IP normalized coverage by the WCE normalized coverage.

Statistical validation of ChIP peaks

After alignment, peaks were called using MACS v2.1.0 [64]. Statistic validation of peaks was performed using the IDR method with ENCODE recommendations [65]. ChIP in a strain h^{90}, *mat1M Δ2-3* and *mat1P Δ2-3* were considered biological replicates for the whole genome since only 10 kb of the silenced donor regions differ. Peaks were called on individual duplicates (h^{90} and *mat1M Δ2-3* sequencing were used) and were called on pseudo-replicates. Pseudo-replicates consist of 2 files that result from merging of biological replicates and their random separation. Irreproducibility Discovery Rate (IDR) was calculated as described by the ENCODE Consortium [65]. The lists of peaks of the biological replicates were challenged with the lists of the pseudo-replicates. This comparison permits to obtain a low IDR threshold that permits to extract the significant peaks (Supplementary Figure S4 and Supplementary Table S1).

5' count analysis and statistical validation

5’ nucleotides were extracted from alignments using a custom script (available upon request). Statistical analysis was performed on a coverage file non-normalized of the 5' count in R. The p-value was calculated using a negative binomial distribution in R (fdist lowtail = FALSE (fitdistrplus)). The theoretical t-student distributions were calculated using the whole genome data (Figure 5 and 6) or on the 10 kb containing *mat1* data (Figure 7 and 8).

Data and R scripts are available upon request. Raw sequencing data will be available on the NCBI database.

SUPPLEMENTAL FIGURES

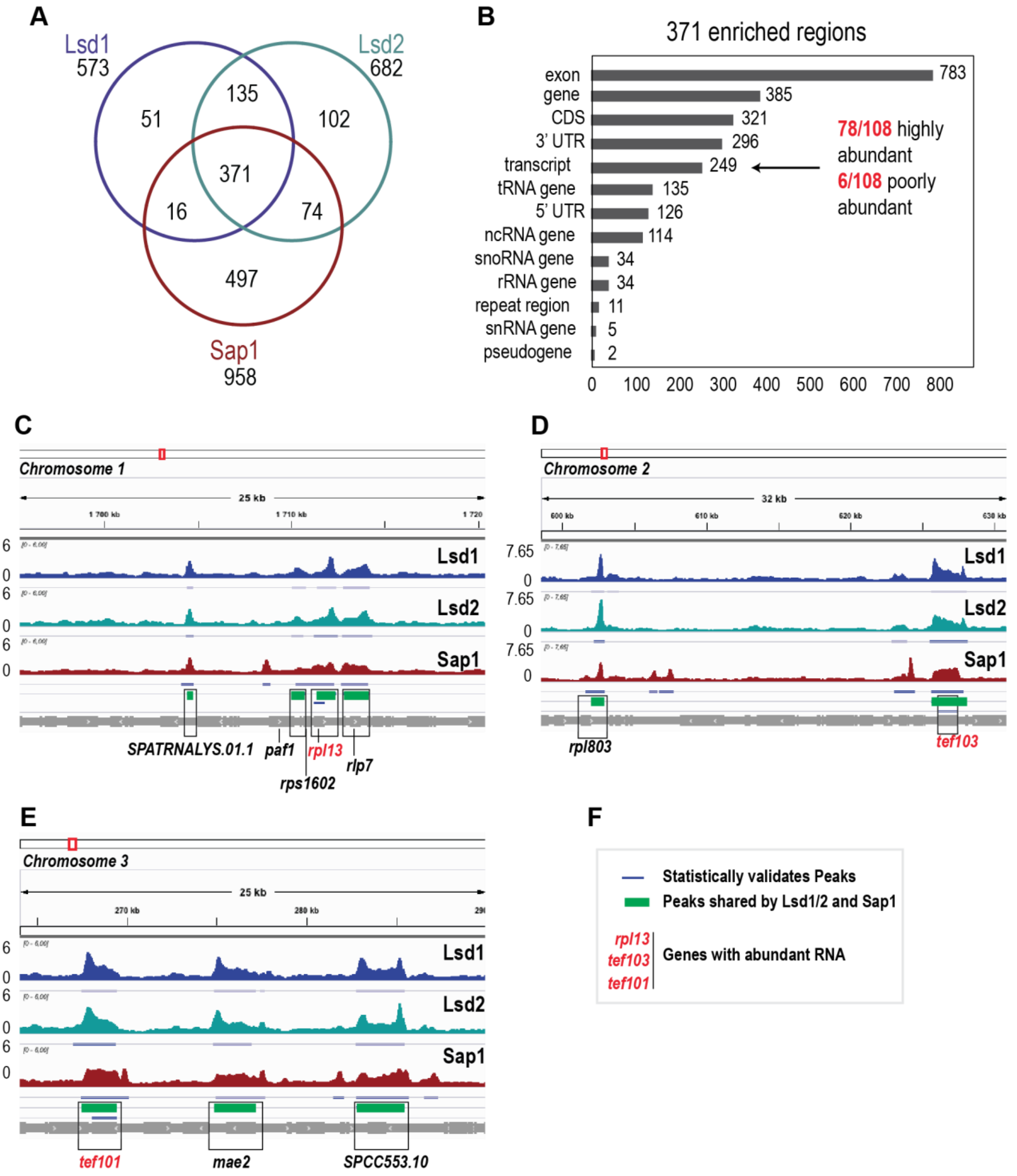

Supplemental FIGURE S1: Lsd1/2 and Sap1 are recruited at the same regions. (A) Overlap of significant enriched peaks of Lsd1, Lsd2 and Sap1. **(B)** Characterization of the 371 peaks enriched in Lsd1/2 and Sap1. Over the 371 peaks 78 are present on 108 highly transcribed gene tested and 6 on 108 poorly transcribed genes tested. Transcriptional data from [67] was used. **(C-E)** Distribution of normalized enrichments of the Lsd1, Lsd2, and Sap1 ChIPs in a h^{90} strain (IP RPkM *reads per kilobase million*/ WCE RPkM). Blue bars indicate significantly enriched peaks. Green bars represent peaks shared by LSD1/2 and Sap1. Genes that are enriched are indicated and in red are highlighted the genes with a high level of transcripts.

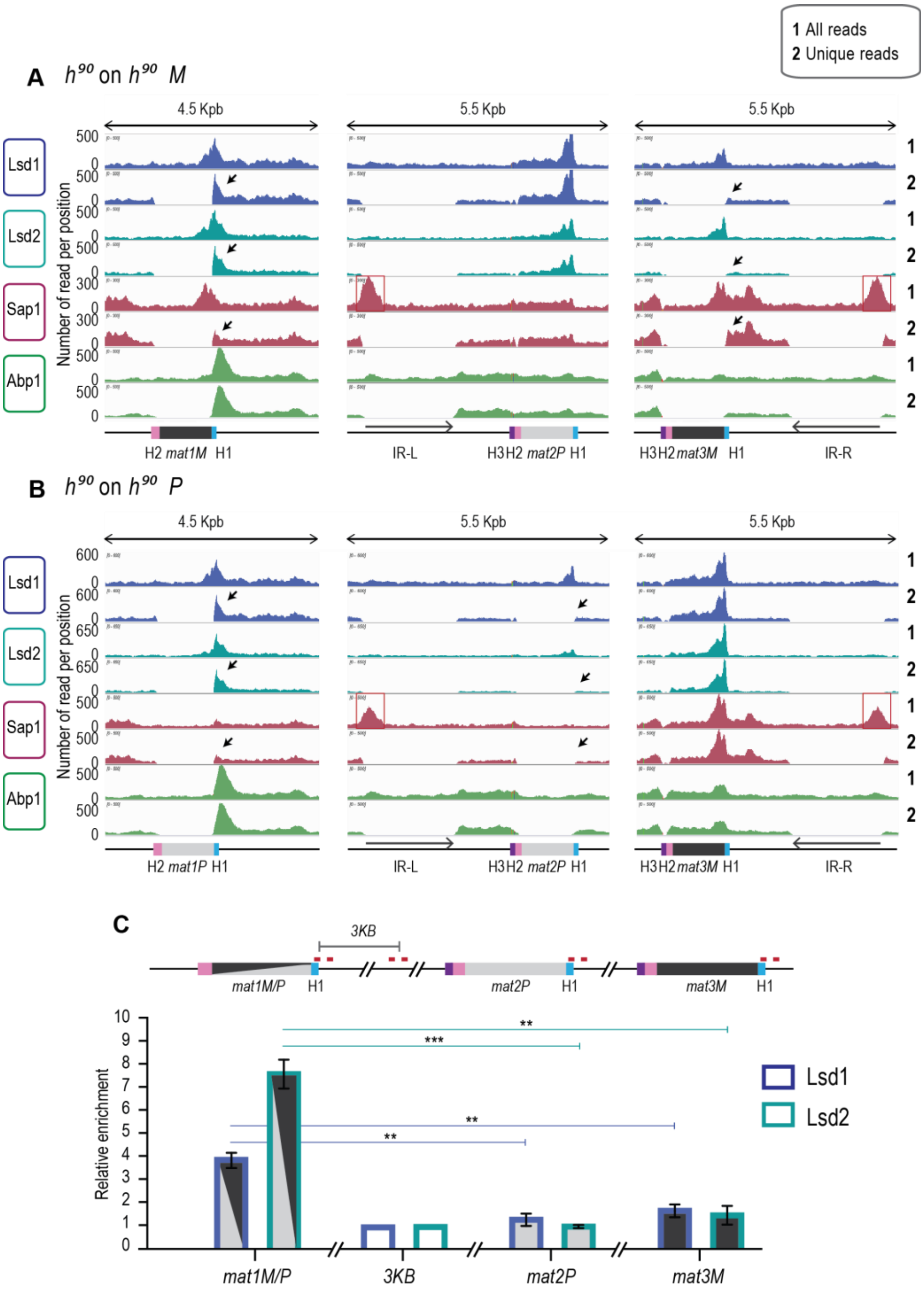

Supplemental FIGURE S2: Lsd1/2 and Abp1 are recruited only at *mat1*. (A) Distribution of raw coverage of the Abp1, Lsd1, Lsd2, and Sap1 ChIPs. The sequence used for the alignment is a 44-kb region that contains the MT region with the *M* allele at *mat1*: h^{90} *M*. The lines numbered 1 are the coverage without filter and the lines numbered 2 are the coverage obtained after removal of multi-mapper reads. The arrows indicate the unique-mapper reads at the junction of the *mat* loci. Red rectangles indicate the Sap1 enrichment at the inverted repeats IR-L and IR-R. **(B)** Same as in A with the h^{90} *P* sequence used for the alignment. **(C)** The upper panel is a schematic view of the MT region. In red, the primer pairs used for the qPCR are annotated. The lower panel shows the result of the ChIP-qPCR of Lsd1 (blue) and Lsd2 (green) at *mat1* (grey and black bar), at 3 kb of *mat1* (white bar), at *mat2P* (grey bar) and at *mat3M* (black bar). The mean of biological triplicates is represented and error bars are the SEM (standard error mean). An unpaired t-test was used to calculate the p value (***<0.005 and **<0.05).

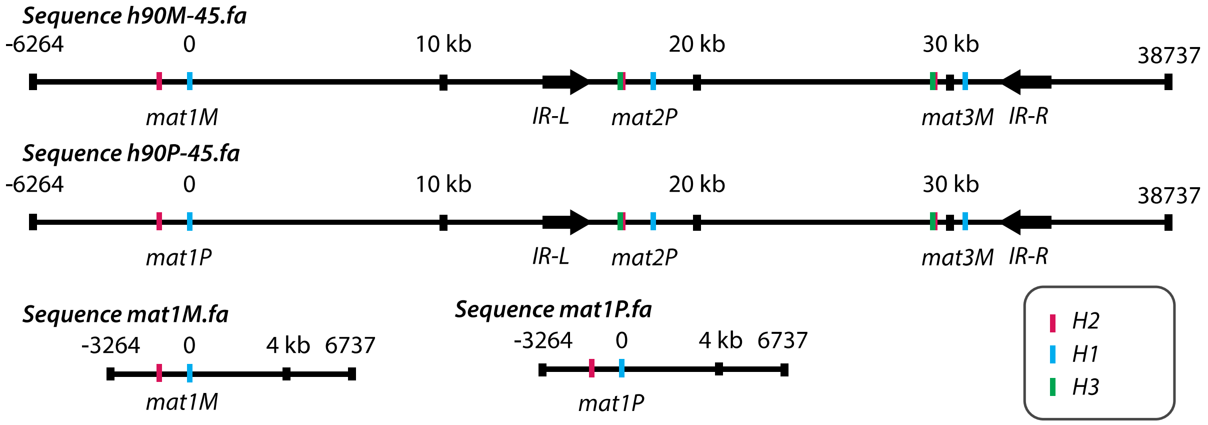

Supplemental FIGURE S3: Contigs used for the alignment. Contigs used for the different alignments are represented. The sequences are available upon request.

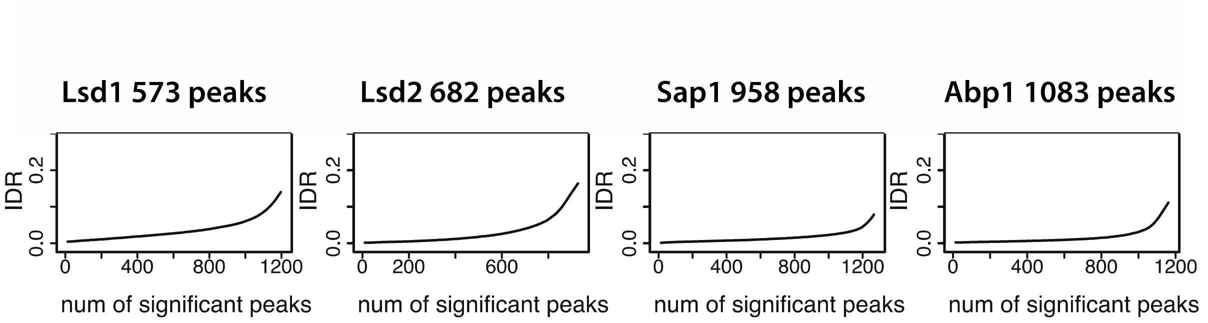

Supplemental FIGURE S4: Result of the IDR analysis. The IDR is presented as a function of the number of significant peaks for Lsd1, Lsd2, Sap1 and Abp1.

SUPPLEMENTAL TABLES

Supplemental TABLE S1: Thresholds obtained after IDR analysis are presented for each immunoprecipitated protein.

	Number of peaks IDR	Threshold = -log10(p-value)
Lsd1	573	10,22646
Lsd2	682	19,19591
Sap1	958	26,07609
Abp1	1083	25,11338

Supplemental TABLE S2: Region enriched in 5'count are indicated.

Peak number	Chr	First enriched 5'	Last enriched 5'	External Name	Logical Name
1	1	71982	72380	SPNUMT.8	nuclear_mt_pseudogne
2	1	3810366	3810458	SPNUMT.17	nuclear_mt_pseudogne
3	2	2115364		Imprint	Imprint
4	2	4532473	4540005	TELII	telomeric region
5	3	0	24683	SPRRNA	rDNA
6	3	1070904	1137003	CENIII	centromeric region
7	3	2439435	2452883	SPRRNA	rDNA

Supplemental TABLE S3: List of strains.

Name	Genotype	Experiment	Origin
PB 70	*mat1M Δ2.3::ScLEU2 ade6-M216 ura- leu1-32*	ChIP	
PB 47	*h90 ade6-M210 leu1-32 uraΔ*	ChIP/ 2D-gel	
PB 2279	*mat1P Δ2.3::ScLEU2 ade6-M216 ura-*	ChIP	
PB 483	*mat1M SS2Δ Δ2.3::ScLEU2 ade6-M210 ura-*	ChIP	
PB 484	*mat1M SS13Δ Δ2.3::ScLEU2 ade6-M210 ura-*	ChIP	
PB 241	*mat1M smt-0 Δ2.3::ScLEU2 ade6-M210 hist2- leu1-32*	ChIP	
PB 2348	*h90 lsd1-Myc::KanMX6 ade6-M210 leu1-32 ura-*	ChIP	Modify from [18]
PB 495	*mat1M Δ2.3::ScLEU2 lsd1-Myc::KanMX6 ade6-M216 ura- leu1-32*	ChIP	
PB 2336	*mat1P Δ2.3::ScLEU2 lsd1-Myc::KanMX6 ade6-M216*	ChIP	
PB 431	*h90 lsd2-Myc::KanMX6 ade6-M210 leu1-32 ura-*	ChIP	
PB 489	*mat1M Δ2.3::ScLEU2 lsd2-Myc::KanMX6 ade6-M216*	ChIP	
PB 2350	*mat1P Δ2.3::ScLEU2 lsd2-Myc::KanMX6 ade6-M216 ura- leu1-32*	ChIP	
PB 1268	*h90 Abp1-CterTAP::KanMX6 ura4-D18 leu1-32*	ChIP	Modify from [33]
PB 1242	*mat1M Δ2.3::ScLEU2 Abp1-CterTAP::KanMX6 leu1-32 ade6-M216*	ChIP	
PB 1257	*mat1P Δ2:3::ScLEU2 Abp1-CterTAP::KanMX6 ade6-M216 ura4-DS/E*	ChIP	
PB 1269	*h90 Abp1-CterTAP::KanMX6 swi1Δ::KAN ade6-M210 leu1-32*	ChIP	
PB 1258	*h90 abp1Δ::ScLEU2 ade6-M210 leu1-32*	2D-gel	
PB 2997	*h90 lsd2-Myc::KanMX6 clr4Δ::KanMX6 ade6-M216 leu1-32*	ChIP	
PB 2995	*h90 lsd1-Myc::KanMX6 clr4Δ::KanMX6 ade6-M216 leu1-32 ura-*	ChIP	

Supplemental TABLE S4: List of sequences of primers used in this study.

Name		Sequence		Experiment
**OL1436 M1+**	**5'**	TATGCTTCTTAGAGTTACATTCACTGAAGATTATAATGTAATATTTTGTG	**3'**	gel-shift
**OL1281 M1-**	**5'**	CACAAAATATTACATTATAATCTTCAGTGAATGTAACTCTAAGAAGCATA	**3'**	
**OL1437 M2+**	**5'**	GAAGATTATAATGTAATATTTTGTGTACCCCATTTGCGTTGAGTTATTCT	**3'**	
**OL1282 M2-**	**5'**	AGAATAACTCAACGCAAATGGGGTACACAAAATATTACATTATAATCTTC	**3'**	
**OL1438 M3+**	**5'**	TACCCCATTTGCGTTGAGTTATTCTATAGTAATTATTGTGTGTTCTATTA	**3'**	
**OL1283 M3-**	**5'**	TAATAGAACACACAATAATTACTATAGAATAACTCAACGCAAATGGGGTA	**3'**	
**OL1439 M4+**	**5'**	ATAGTAATTATTGTGTGTTCTATTAACGATGTATTGCGATTTATATCTGT	**3'**	
**OL1284 M4-**	**5'**	ACAGATATAAATCGCAATACATCGTTAATAGAACACACAATAATTACTAT	**3'**	
**OL1440 M5+**	**5'**	ACGATGTATTGCGATTTATATCTGTTATGCTAACATAACGTAGTTCTAAG	**3'**	
**OL1285 M5-**	**5'**	CTTAGAACTACGTTATGTTAGCATAACAGATATAAATCGCAATACATCGT	**3'**	
**OL1441 M6+**	**5'**	TATGCTAACATAACGTAGTTCTAAGCACTGTAATGCCATACTGTTTTAGA	**3'**	
**OL1286 M6-**	**5'**	TCTAAAACAGTATGGCATTACAGTGCTTAGAACTACGTTATGTTAGCATA	**3'**	
**OL1442 M7+**	**5'**	CACTGTAATGCCATACTGTTTTAGAGGGTGATGCTTCCTAAAATCTCCTT	**3'**	
**OL1287 M7-**	**5'**	AAGGAGATTTTAGGAAGCATCACCCTCTAAAACAGTATGGCATTACAGTG	**3'**	
**OL1443 M8+**	**5'**	GGGTGATGCTTCCTAAAATCTCCTTACATAAAGTAATACATGGATTTTAC	**3'**	
**OL1288 M8-**	**5'**	GTAAAATCCATGTATTACTTTATGTAAGGAGATTTTAGGAAGCATCACCC	**3'**	
**OL1444 M9+**	**5'**	ACATAAAGTAATACATGGATTTTACTGCCCTGATTCTATCGAAATATGCT	**3'**	
**OL1289 M9-**	**5'**	AGCATATTTCGATAGAATCAGGGCAGTAAAATCCATGTATTACTTTATGT	**3'**	
**OL1445 M10+**	**5'**	TGCCCTGATTCTATCGAAATATGCTGTTTTTTTTATTCGTTTTTATTTAT	**3'**	
**OL1290 M10-**	**5'**	ATAAATAAAAACGAATAAAAAAAACAGCATATTTCGATAGAATCAGGGCA	**3'**	
**OL1446 H1+**	**5'**	TTTGTAATATAAATGTATAGTCTTTCTCCTTTGTTTTCTCTCGTTCGTTT	**3'**	
**OL1291 H1-**	**5'**	AAACGAACGAGAGAAAACAAAGGAGAAAGACTATACATTTATATTACAAA	**3'**	
**OL1447 SAS1+**	**5'**	CTCTAACGAGATATTTGCTTCGCTACGCTACG	**3'**	
**OL1448 SAS1-**	**5'**	CGTAGCGTAGCGAAGCAAATATCTCGTTAGAG	**3'**	
**OL1449 D1+**	**5'**	TTCTCTCGTTCGTTTCCATGTTTCCAATTATG	**3'**	
**OL1292 D1-**	**5'**	CATAATTGGAAACATGGAAACGAACGAGAGAA	**3'**	
**OL1492 Ter1+**	**5'**	GGGATTTAACGCAGTGCAAGGAGCTATCTTGG	**3'**	
**OL1493 Ter1 -**	**5'**	CCAAGATAGCTCCTTGCACTGCGTTAAATCCC	**3'**	
**probe mat1**	**5'**	TTCGGTATTTAAGTCTGGCG	**3'**	2D gel
**probe mat1**	**5'**	CCAATTATGCTGTTCGTGTC	**3'**	
**OL194 H1**	**5'**	CTCCTTTGTTTTCTCTCGT	**3'**	qPCR
**OL195 mat1**	**5'**	TGGTTGATGGAGTGGTTG	**3'**	
**OL196 mat2**	**5'**	CTTCGTGGTATTCGGAAAT	**3'**	
**OL1524 mat3**	**5'**	CTCCTTTGTTTTCTCTC	**3'**	
**OL1525 mat3**	**5'**	ATGTTGGCAAAACGA	**3'**	
**OL280 3kb**	**5'**	CTCCCATCCTTGTCCTTT	**3'**	
**OL281 3kb**	**5'**	GGCGCTCATGGTTATCTT	**3'**	

Supplemental TABLE S5: Libraries statistics.

Name	Library	Run Type	Total Reads	Aligned Reads	% Aligned Reads
PB 2348	h90lsd1IP	SR65	19681930	18482514	93.91%
PB 2348	h90lsd1WCE	SR65	21067533	19342359	91.81%
PB 431	h90lsd2IP	SR65	8988245	8152554	90.70%
PB 431	h90lsd2WCE	SR65	47617742	44206781	92.84%
PB 47	h90sap1IP	SR65	12951987	12459329	96.20%
PB 47	h90sap1WCE	SR65	62223348	55091901	88.54%
PB 1268	h90abp1IP	SR130	6462438	5690753	88.06%
PB 1268	h90abp1WCE	SR130	46506268	40369759	86.80%
PB 495	mat1Mlsd1IP	SR65	78402964	70328896	89.70%
PB 495	mat1Mlsd1WCE	SR65	43201409	39627677	91.73%
PB 489	mat1Mlsd2IP	SR65	10184098	9714898	95.39%
PB 489	mat1Mlsd2WCE	SR65	9408560	8715332	92.63%
PB 70	mat1Msap1IP	SR65	12714852	12095278	95.13%
PB 70	mat1Msap1WCE	SR65	71445515	60200476	84.26%
PB 1242	mat1Mabp1IP	SR130	11015722	9764199	88.64%
PB 1242	mat1Mabp1WCE	SR130	42626692	37576928	88.15%
PB 2336	mat1Plsd1IP	SR65	36559765	33949628	92.86%
PB 2336	mat1Plsd1WCE	SR65	25466207	23759984	93.30%
PB 2350	mat1Plsd2IP	SR65	9443782	9170279	97.10%
PB 2350	mat1Plsd2WCE	SR65	30848920	28570601	92.61%
PB 2279	mat1Psap1IP	SR65	9003795	8611021	95.64%
PB 2279	mat1Psap1WCE	SR65	15378877	13342280	86.76%
PB 1257	mat1Pabp1IP	SR130	36287760	32457178	89.44%
PB 1257	mat1Pabp1WCE	SR130	78169993	35976808	46.02%
PB 483	mat1MSS2sap1IP	SR65	6003899	5124804	85.36%
PB 483	mat1MSS2sap1WCE	SR65	52438070	46350961	88.39%
PB 484	mat1MSS13sap1IP	SR65	9870989	9139481	92.59%
PB 484	mat1MSS13sap1WCE	SR65	46317419	40703242	87.88%
PB 241	mat1Msmtsap1IP	SR65	5074834	4777575	94.14%
PB 241	mat1Msmtsap1WCE	SR65	35281724	31718229	89.90%
PB 1269	h90abp1swi1IP	SR130	6868375	5599274	81.52%
PB 1269	h90abp1swi1WCE	SR130	28327346	22514091	79.48%
